# Lipid Processing in the Brain: A Key Regulator of Systemic Metabolism

**DOI:** 10.3389/fendo.2017.00060

**Published:** 2017-04-04

**Authors:** Kimberley D. Bruce, Andrea Zsombok, Robert H. Eckel

**Affiliations:** ^1^University of Colorado School of Medicine, Division of Endocrinology, Metabolism and Diabetes, Aurora, CO, USA; ^2^Department of Physiology, School of Medicine, Tulane University, New Orleans, LA, USA

**Keywords:** lipid metabolism, brain, liver, energy homeostasis, hypothalamus

## Abstract

Metabolic disorders, particularly aberrations in lipid homeostasis, such as obesity, type 2 diabetes mellitus, and hypertriglyceridemia often manifest together as the metabolic syndrome (MetS). Despite major advances in our understanding of the pathogenesis of these disorders, the prevalence of the MetS continues to rise. It is becoming increasingly apparent that intermediary metabolism within the central nervous system is a major contributor to the regulation of systemic metabolism. In particular, lipid metabolism within the brain is tightly regulated to maintain neuronal structure and function and may signal nutrient status to modulate metabolism in key peripheral tissues such as the liver. There is now a growing body of evidence to suggest that fatty acid (FA) sensing in hypothalamic neurons *via* accumulation of FAs or FA metabolites may signal nutritional sufficiency and may decrease hepatic glucose production, lipogenesis, and VLDL-TG secretion. In addition, recent studies have highlighted the existence of liver-related neurons that have the potential to direct such signals through parasympathetic and sympathetic nervous system activity. However, to date whether these liver-related neurons are FA sensitive remain to be determined. The findings discussed in this review underscore the importance of the autonomic nervous system in the regulation of systemic metabolism and highlight the need for further research to determine the key features of FA neurons, which may serve as novel therapeutic targets for the treatment of metabolic disorders.

## Introduction

Metabolic disorders, particularly aberrations in lipid homeostasis, such as obesity, type 2 diabetes mellitus (T2D), non-alcoholic fatty liver disease, and hypertriglyceridemia, often manifest together as the metabolic syndrome (MetS) (1). Despite major advances in our understanding of the pathogenesis of these disorders, the prevalence of the MetS continues to rise (2). Since MetS constitutes an increased risk to cardiovascular morbidity and mortality (3), a more detailed understanding of the common causes, and integration between these disorders of energy homeostasis, is necessary to identify novel therapeutic targets and interventions that may halt the development of severe metabolic disease.

It is becoming increasingly apparent that the central nervous system (CNS) is a major contributor to the regulation of systemic metabolism and lipid balance. In the CNS, the nutritional status of the body is constantly being surveyed and assessed by key energy-sensing regions of the brain, such as the hypothalamus. Key nuclei within the hypothalamus, such as the ventromedial nucleus (VMH), arcuate nucleus (ARC), dorsomedial hypothalamic nucleus (DMH), and the paraventricular nucleus (PVN), integrate signals to elicit peripheral responses, such as changes in feeding behavior, fuel mobilization, energy utilization, and energy storage (4). These nuclei detect both nutrients and nutritionally regulated endocrine factors, such as insulin (5), ghrelin (6), melanocortin (MC) (7), and leptin (8), in order to regulate feeding and energy balance. Here, in this review, we will focus on the mechanisms involved in lipid sensing in the brain and its emerging influence on systemic metabolism.

## How Do Lipids Enter the Brain?

Lipids and lipid intermediates are essential components of the structure and function of the brain. In fact, the brain has the second highest lipid content behind adipose tissue, and brain lipids constitute 50% of the brain dry weight (9). However, unlike adipose tissue, which largely stores FAs as triglycerides for subsequent utilization and mobilization to other metabolic tissues, the brain is thought to mainly utilize acylated lipids to generate phospholipids for cell membranes (9). The FA composition of the brain is unique and is rich in long-chain polyunsaturated fatty acids (LC-PUFAs), particularly arachidonic acid (AA), eicosapentaenoic acid, and docosahexaenoic acid (DHA). Although some FAs can be synthesized *de novo*, essential FAs must be transported into the brain from the systemic circulation. And contrary to previously held theories, recent data suggest that this is a dynamic process, with up to 8% of LC-PUFAs actively being turned over daily and being replaced by plasma-derived FAs (10). Indeed, a number of studies have shown that FAs are able to cross the blood–brain barrier (BBB) and enter neurons. For example, radiolabeled FAs that are injected into the carotid artery of rats can be traced to neuronal cells (10). In addition, brain perfusion studies in rats have shown that radiolabeled palmitate is readily incorporated into cerebral phospholipids and neutral lipids and has a similar rate of uptake when delivered *via* whole rat plasma or a synthetic saline containing physiological levels of albumin. This suggests that FAs cross the BBB and that albumin may be important in this process (11). Even though these studies convincingly demonstrate FA uptake, they do not address the mechanism of transport.

How FAs enter the brain remains an unanswered fundamental question. Although we cannot rule out the possibility that FAs could passively diffuse across the BBB, several studies highlight the role of FA transporters in this process. The membrane localized FA transport proteins (FATP1 and FATP4) appear to be the predominant FA transport proteins expressed in the BBB based on human and mouse expression studies, whereas the FA translocase/CD36 plays a prominent role in the transport of FAs across human brain microvessel endothelial cells (12). Furthermore, cytosolic-localized fatty acid-binding protein 5 has an important function in FA transport across cultured brain microvascular cells (13). Although still under active investigation, is it plausible that some transporters may also exhibit specificity toward particular FAs. For example, the major facilitator superfamily d 2 a, which is exclusively expressed in the endothelium of the BBB, has been shown to selectively transport DHA in the form of the partially hydrolyzed phospholipid, lysophosphatidylcholine, otherwise known as lysolecithin (14).

## Neuronal Uptake of Lipids and FAs

Neuronal uptake of FAs remains an important yet poorly understood process. However, it is possible that once FAs have traversed the BBB, FA transporters may also play a key role in facilitating FA uptake into neurons. For example, dissociated neurons from the VMH express both FATP1 and CD36 (15). Specifically, important studies using fura-2 calcium imaging and fluorometric imaging plate reader membrane potential dye, in addition to pharmacological manipulations have shown that while neurons of the VMH and ARC respond to oleic acid (OA, C18:1 n-9) (15, 16), this response is lost when CD36 is depleted in the VMH using an adeno-associated viral (AAV) vector expressing CD36 short hairpin RNA (17). Since CD38 is an established gustatory lipid sensor, it is also plausible that other lipid sensors involved in the chemoreception of long-chain fatty acids (LCFAs)/omega-3 fatty acids (ω-3 FAs), such as GPR120, are also involved in neuronal lipid sensing. However, although GPR120 is functionally active in immortalized hypothalamic neurons and mediates the anti-inflammatory actions of the ω-3 FA, DHA (18), its role in neuronal lipid sensing *in vivo* has not been determined. In addition, FABP3, which is localized in neurons, facilitates brain AA but not palmitic acid (C16:0) uptake and trafficking into specific brain lipid pools (19).

It is also becoming more widely accepted that neurons may receive metabolic support from glial cells in a variety of forms. The “astrocyte–neuron lactate shuttle” has been postulated, whereby astrocytes metabolize glucose to release lactate, which is then taken up by the neuronally expressed monocarboxylate transporter, providing a supplemental energy source for neurons (20). Moreover, lipid metabolism in astrocytes plays a key role in FA sensing, since FA oxidation is thought to occur predominantly in the astrocyte rather than the neuron. When the levels of FAs are increased, as in the case of a high-fat diet (HFD), astrocyte-mediated lipid oxidation results in elevated ketone levels in the brain. Conversely, changes in ketone abundance within energy-sensing regions of the hypothalamus are able to modify energy homeostasis. Specifically, reduced ketone production within the VMH and ARC signals a decrease in HFD intake, and ketone production can override glucose and FA sensing in VMH neurons (21, 22). Astrocytes may play a key role in the regulation of energy balance by sensing LCFAs as metabolic signals. For example, hypothalamic but not cortical astrocytes have a high capacity for the oxidation of LCFAs (23), and this flux may be AMP-activated protein kinase (AMPK) dependent. Both neurons of the VMH and astrocytes have been shown to express many of the FA transporters, including FATP1, FATP4, and CD36. Most recently, the fatty acid bind protein 7 (FABP7) was shown to be important for astrocyte–neuron lipid homeostasis. Mice lacking FABP7 develop neuropsychiatric disorders such as schizophrenia, which may at least in part be due to aberrant dendritic spine morphology, and decreased spine density compared to WT mice (24). Moreover, transplantation of WT astrocytes into FABP7 KO mice partially attenuated cognitive impairments (24). Once transported into the cell, LCFAs are esterified by acyl-CoA-binding protein (ACBP), a protein that is ubiquitously expressed in tissues with high lipid turnover. Interestingly, ACBP is expressed in the hypothalamus and may play a role in the LCFA sensing by hypothalamic astrocytes (25).

Astrocytes are also critical for the regulation of cholesterol homeostasis in the neuron. Cholesterol is an essential component of neuronal physiology during both development and adulthood and is independently tightly regulated in the brain, largely due to the existence of the BBB (26). Cholesterol depletion in neurons impairs vital functions, including synaptic vesicle exocytosis, neuronal activity, and neurotransmission and results in synaptic loss and neurodegeneration (27, 28). Clinically, deficits in cholesterol homeostasis in the CNS manifest as severe primary neurological disorders such as Neimann–Pick C disease and Parkinson’s disease (26). It is thought that astrocytes are a major site of lipoprotein synthesis and assembly in the brain (29). Of particular relevance is apolipoprotein E (ApoE), a 39-kDa protein that is highly expressed in the brain, surpassed only by hepatic ApoE production (30).

Apolipoprotein E-containing lipoproteins have been extensively studied and are known to have several major functions. For example, ApoE-containing high-density lipoprotein (HDL)-like lipoproteins are secreted by astrocytes and are taken up into neurons *via* low-density lipoprotein receptors. This transfer of key lipids and cholesterol facilitates axonal extension and neuronal survival and requires the presence of sphingomyelin in the ApoE-containing lipoprotein particle (31, 32). ApoE does not only play a major role in glia-neuronal lipid metabolism but also acts as a ligand for multiple receptors in neurons, which interact with a number of downstream physiological processes (33). In particular, a number of recent studies have shown that brain ApoE is an important regulator of peripheral energy homeostasis. In rats, intracerebroventricular (ICV) ApoE injections significantly decreased food intake, whereas infusion of ApoE antiserum stimulated feeding, therefore suggesting that ApoE may be important satiety factor in the hypothalamus (34). In support, exogenous ApoE treatment has been shown to activate phosphatidylinositol-3-kinase (PI3K)/Akt signaling, and PI3K inhibition by LY294002 attenuates both ApoE-induced signaling and satiation (35).

Apolipoprotein E has three major isoforms (ApoE2, ApoE3, and ApoE4), which have varying effects on lipid homeostasis and neuronal function. While both ApoE2 and ApoE3 preferentially associate with phospholipid rich HDL-like particles, ApoE4 prefers large triglyceride-rich VLDL particles (36). Interestingly, ApoE4 has been repeatedly implicated in the pathogenesis of Alzheimer’s disease (AD) and may bind to the Aβ peptide leading to impair Aβ clearance (37). Current hypothesis suggests that the proteolytic cleavage products of ApoE4 may have a central role in AD pathology, since AD patients have markedly increased levels of these cleavage products compared to controls (38).

These findings suggest that lipoprotein metabolism in the CNS may be key to lipid homeostasis and nutrient sensing; however, to date, the molecular mechanisms underlying these processes remain largely unknown. Nonetheless, we have recently shown that lipoprotein lipase (LPL), the rate-limiting enzyme in the hydrolysis of lipoprotein-derived FAs, may facilitate the uptake of FAs into dissociated hypothalamic neurons (39). Moreover, mice with a specific neuronal LPL deficiency exhibit a defect in nutrient sensing and become hyperphagic, relatively inactive and obsessed compared to WT control mice (40). Interestingly, these mice are also polyunsaturated fatty acid (PUFA) deficient in the hypothalamus (40), and in the hippocampus, where LPL deficiency is also associated with alterations in learning and memory and synaptic function (41). In further support, a specific deletion of LPL in the hippocampus also leads to increased weight gain and decreased activity *via* a ceramide-dependent pathway (42). We have also shown that while mice lacking pan-neuronal LPL develop obesity, this obesity is not exacerbated on an HF diet, or rescued by a PUFA enriched diet, further highlighting the importance of LPL in lipid sensing and body weight regulation (43). These data highlight the potential role of LPL as a mechanistic link between brain lipid uptake, neuronal lipoprotein metabolism, and nutrient sensing; however, the precise role of LPL in lipid sensing in both neurons and glia requires further investigation.

## Hypothalamic FA Metabolism

The evidence for facilitated FA uptake into neurons is limited, which may be due to the long-held view that neurons do not derive much of their energy supply from lipids. However, neurons do express many molecular components of lipid catabolism pathways, suggesting that lipid utilization is a critical process to neuronal function. For example, neurons of the VMH express enzymes involved in the intracellular metabolism of FAs, including long-chain acyl-CoA synthase (ACS), carnitine palmitoyltransferase-1a and 1c (CPT-1a and 1c), and uncoupling protein-2 (UCP2), and enzymes involved in *de novo* lipogenesis, such as fatty acid synthase (FAS) (15). In addition, the nuclear receptor peroxisome proliferator-activated receptor (PPARγ), a key factor in lipid metabolism that can be activated by endogenous lipid ligands to promote adipogenesis and insulin sensitivity, is predominantly expressed in neurons of the hypothalamus (44), including the ARC.

Activation of these intracellular metabolic pathways in hypothalamic cells in response to FAs provides further support of the role of lipid metabolism and sensing in hypothalamic neurons. For example, UCP2, which increases proton leak from the respiratory electron transport chain, may act as a metabolic switch from glucose metabolism to mitochondrial FA oxidation in hypothalamic NPY/AgRP neurons during fasting (45). Interestingly, UCP2 likely mediates the actions of ghrelin, which is increased upon fasting, on the activation of NPY/AgRP neurons. While ghrelin increases palmitate-induced mitochondrial respiration, this is not observed in UCP2−/− mice. Moreover, NPY/AgRP neurons of UCP2−/− mice do not show the ghrelin-induced increase in mitochondrial biogenesis (45). In normal circumstances, increased FA oxidation increases reactive oxygen species (ROS), which are then scavenged by UCP2 *via* enhanced proton leak. However, in UCP2−/− mice, these ROS levels remain increased, supporting the hypothesis that ghrelin-triggered ROS production promotes UCP2 activity and mRNA expression to further promote ROS scavenging (45). These studies also suggest that ROS may be involved in neuronal lipid metabolism and activation. In further support, suppression of ROS activates NPY/AgRP neurons to promote feeding, whereas activation of ROS activates proopiomelanocortin (POMC) neurons to reduce feeding. Although further studies are warranted, it is likely that neuronal ROS accumulation may intrinsically link neuronal substrate metabolism to feeding behavior and systemic energy balance (46).

AMP-activated protein kinase may also act as a cellular energy sensor within neurons to link neuronal lipid metabolism to systemic lipid metabolism and energy balance. AMPK is widely expressed in the ARC, PVN, and VMH of the hypothalamus and is able to sense intracellular energy status by the AMP/ATP ratio and the level of adipokines (e.g., leptin and ghrelin) [see Ref. (47) for a comprehensive review]. Activated AMPK responds to the cellular energy status and can switch cellular metabolism toward catabolic processes that produce ATP and away from anabolic processes that consume ATP (48). In response to glucose, hypothalamic AMPK activity is inhibited leading to the activation of acetyl-CoA carboxylase (ACC) and the generation of malonyl-CoA from glucose-derived acetyl-CoA, with downstream effects on food intake (described in more detail below) (49). Similar to peripheral tissues, malonyl-CoA is thought to inhibit CPT-1a and LCFA oxidation in the brain (49). Specifically, glucose inhibits palmitate oxidation *via* AMPK in hypothalamic neurons (23). Hypothalamic AMPK may also regulate downstream lipid metabolism through brown adipose tissue (BAT) thermogenesis. A number of recent studies have suggested that hypothalamic AMPK is involved in the autonomic regulation of BAT thermogenesis, specifically the SNS. For example, central administration of 3,3′,5′-triiodothyronine (T_3_) within the VMH stimulates a thermogenic response associated with decreased AMPK activity in the VMH and elevated sympathetic firing in BAT (50). Whether this effect is observed following neuronal glucose (or lipid) sensing remains to be seen. Nonetheless, this interesting topic has recently been reviewed in detail (51).

AMP-activated protein kinase also plays a key role in the hypothalamic response to leptin in the context of high-fat feeding. While leptin results in reduced food intake and reduced hypothalamic AMPK activity, these effects were not observed in diet-induced obese mice (52). Hypothalamic phospho-AMPK is also modulated by the FA composition of the diet and is dependent on brain region and metabolic status (53). Interestingly, AMPK regulates the activity of a number of enzymes involved in the synthesis of complex lipids that are critical for optimal brain function and metabolism. For example, long-term AMPK stimulation blunted FA-mediated induction of serine palmitoyl transferase and the synthesis of ceramides *de novo* and has thus been shown to protect against fatty acid-mediated apoptosis in the astrocyte (54). Similarly, AMPK is highly expressed in neurons due to their high-energy demands and can promote neuronal survival during periods of glucose deprivation (55). Recently, neuronal AMPK has also been implicated in the pathogenesis of AD. AMPK activity can reduce sphingomyelin levels, inhibit Aβ generation, and reduce amyloid precursor protein (APP) distribution in lipid rafts, whereas deletion of AMPKα2 increases sphingomyelin and APP distribution in lipid rafts (56).

There is growing evidence to suggest that the accumulation of FA metabolites may signal nutrient status and thus may be critical to central lipid sensing, and the modulation of systemic metabolism. For example, upon entry into the neuron, LCFAs are esterified to LCFA-CoA by ACS. It is thought that this accumulation of FA derived LCFA-CoA triggers a lipid-sensing mechanism to inhibit hepatic glucose production (HGP) and to maintain systemic glucose homeostasis (57). In support, direct inhibition of ACS in the hypothalamus disrupts the accumulation of hypothalamic LCFA-CoA, and in turn disrupts the inhibitory effect on hepatic gluconeogenesis, resulting in dysregulated glucose production (58).

In metabolic tissues, intracellular LCFA-CoAs enter the mitochondria *via* CPT-1, where they are then subject to FA β-oxidation. Importantly, the liver isoform of CPT-1, CPT-1a, is prevalent in the hypothalamus, and inhibition of hypothalamic CPT-1a causes an increase in intracellular LCFA-CoA, which triggers a satiation signal, leading to reduced systemic glucose production and food intake (59). However, the neurocircuitry is complex since the role of CPT-1a may vary between hypothalamic nuclei. For example, VMH-selective overexpression of CPT-1a causes over feeding, a phenotype which can be reversed with the CPT-1 specific inhibitor etomoxir (60). In support, long-term overexpression of permanently activated CPT-1a using a viral AAV vector injected into the VMH of rats, leads to hyperghrelinemia, increased food intake, increased body weight, hyperglycemia, and insulin resistance (61). In contrast, CPT-1a expression in the ARC does not have the same effect on the central regulation of feeding (60). Interestingly, the brain also expresses a neuron-specific isoform of CPT-1, CPT-1c, which is found in the endoplasmic reticulum (ER) of key energy-sensing nuclei of the hypothalamus (ARC) and has also been repeatedly implicated in the modulation of systemic metabolism (62–64). Specifically, CPT-1c KO mice have reduced body weight and food intake compared to control mice (62, 63). CPT-1c does not have a typical acyltransferase activity, and thus its precise molecular function is less well understood. Nonetheless, data from recent studies suggest that the orexigenic action of ghrelin is associated with increased hypothalamic (C18:0) ceramide levels, an effect that is blunted in CPT-1c KO mice (65). While the mechanism linking increased ceramide levels to energy balance remain an active area of research, recent studies have demonstrated that ceramides induce hypothalamic lipotoxicity and ER stress, which leads to sympathetic inhibition, reduced BAT thermogenesis, weight gain, and hepatic steatosis (66). In addition, recent metabolomic analysis of the brains of CPT-1c KO mice shows reduced levels of oxidized glutathione, suggesting that CPT-1c may play a role in neuronal oxidative metabolism (67). In addition, these mice show suppressed endocannabinoid levels, which offers an alternative yet consistent mechanism to account for the suppressed food intake observed in CPT-1c KO mice (67). In addition, to their established role in appetite modulation, endogenous endocannabinoids may serve as functional neuromodulatory lipids, derived from neuronal phospholipids, which once secreted undergo lipid catabolism within glial cells. A detailed description of endocannabinoid signaling is beyond the scope of this manuscript but has been previously reviewed in depth (68).

CPT-1 has been suggested to act downstream of malonyl-CoA in the hypothalamic control of feeding (60). This is particularly pertinent to FA metabolism in hypothalamic neurons since CPT-1 activity is inhibited by malonyl-CoA (69), and thus elevated malonyl-CoA leads to an accumulation of LCFA-CoA (70), which has been previously referred to as a satiety signal. Indeed, manipulation of the key enzymes involved in malonyl-CoA metabolism, including ACC, FAS, and malonyl-CoA decarboxylase (MCD), have all been shown to have major effects on food intake and peripheral metabolism (71). For example, ACC, which catalyzes the carboxylation of acetyl-CoA to malonyl-CoA, is expressed in the ARC of the hypothalamus, where it is increased following central leptin treatment resulting in elevated malonyl-CoA and reduced food intake (72). Similarly, inhibition of FAS activity by central administration of the pharmacological FAS inhibitor C75 has been shown to reduce food intake (73) and to elevate malonyl-CoA levels (74). MCD, which is important for malonyl-CoA degradation, is also important for regulating lipid intermediates and systemic metabolism. Overexpression of MCD in the mediobasal hypothalamus (MBH) chronically reduces malonyl-CoA levels and causes rapid increases in food intake and weight gain (75). In addition, MBH expression depleted both malonyl-CoA and LCFA-CoA and was sufficient to induce hepatic insulin resistance in the presence of hyperlipidemia (75). These studies begin to highlight the importance of hypothalamic sensing of circulating lipids in the maintenance of hepatic metabolism and systemic glucose homeostasis. However, the physiological and molecular mechanisms that integrate the brain–liver axis remain unresolved and an active area of investigation.

## Hypothalamic Lipid Sensing and Hepatic Metabolism

Early studies by Obici and colleagues, where OA was administered into the brain *via* ICV injection, were among the first to demonstrate the profound central effects of LCFAs on peripheral metabolism. Interestingly, the short chain FA octanoic acid (C8) did not have the same effect. Moreover, these studies showed that central OA administration could lower plasma insulin and glucose levels under basal physiological conditions. To determine the mode of blood glucose reduction, pancreatic euglycemic clamps were performed during ICV infusion of OA. ICV OA administration leads to a marked decline in glucose production compared to basal levels (59). However, when clamps were performed in the presence of ICV OA and sulfonylurea, a potent inhibitor of neuronal K_ATP_ channels, the profound effect of central OA on HGP was blunted, suggesting that OA acutely enhances hepatic insulin action *via* the activation of K_ATP_ channels in the hypothalamus (59). Further studies support this notion and have shown that pharmacological activation of K_ATP_ channels in the hypothalamus (*via* hypothalamic diazoxide administration) suppresses HGP (76). These observations, taken together with the findings from CPT-1 inhibition studies, strongly suggest that the accumulation of LCFA-CoAs is able to suppress HGP (70). Recent studies have suggested that this system may actually be even more complex and have shown that the effect of LCFAs on hepatic metabolism may be FA specific. For example, while bilateral infusion of OA into the MBH results in a significant reduction in HGP, palmitic acid (PA, C16:0) has a lesser effect, and linoleic acid has no effect compared to vehicle control (LA, C18:2 n-6) (77). These data suggest that mono-unsaturated fatty acids are a more potent suppressor than saturated fatty acids or PUFAs; however, the mechanisms underlying these differential effects of FAs on hepatic glucose metabolism remain to be determined.

In addition to the central regulation of HGP, hypothalamic nutrient sensing may also be a key regulator of hepatic lipid homeostasis. The liver maintains lipid homeostasis through tightly coordinated synthesis and secretion of triglyceride-rich lipoproteins (VLDL-TG), lipogenesis, and FA oxidation. Interestingly, infusion of the orexigenic neuropeptide Y directly into the third ventricle of the hypothalamus has been shown to increase hepatic VLDL-TG secretion, which may be part of the physiological response to fasting when lipids become the main energy source and NPY neurons in the ARC of the hypothalamus are activated (78). Thus, VLDL-TG secretion may be a response by the autonomic nervous system (ANS) to mobilize lipids during a period of relative nutrient deficiency. In addition, sympathetic denervation prevented the increase in the VLDL-TG secretion in the fasted state, whereas total denervation, or parasympathetic denervation did not, suggesting that the central regulation of hepatic lipid mobilization during fasting may be largely mediated through the sympathetic nervous system (78). In further support, sympathetic hepatic denervation also prevented the stimulatory effect of NPY on VLDL-TG secretion (78). This is in contrast to glucose sensing in the MBH (79), and glycine in the dorsal vagal complex (DVC) (80), which is thought to signal sufficient nutrient status and inhibit VLDL-TG secretion, possibly through the parasympathetic nervous system.

In addition to NPY, a number of studies have also shown that MC expressing neurons of the hypothalamus may also regulate hepatic lipogenesis and TG metabolism. Central administration of MTII, a synthetic MC3/4 receptor agonist, has been shown to reduce hepatic lipogenic gene expression in mice (81) and decrease hepatic TG content in rats (82), suggesting that increased MC signaling can inhibit hepatic VLDL-TG production. In support of this notion, central administration of an MC3/4 receptor antagonist, markedly increased liver TG content in rats, strongly suggesting that hepatic lipogenesis was increased (7).

Melanin-concentrating hormone (MCH) is also involved in the neuronal circuits that modify autonomic outflow to the liver and white adipose tissue. MCH-deficient mice are hyperphagic and lean when fed a normal diet (83), are resistant to age associated insulin resistance (84), and when fed an HFD they are resistant to obesity and hepatic steatosis (85). Recent key studies have also shown that genetic activation of MCH specifically in the LH triggers hepatic lipid accumulation and lipoprotein uptake (86).

**Figure 1:**
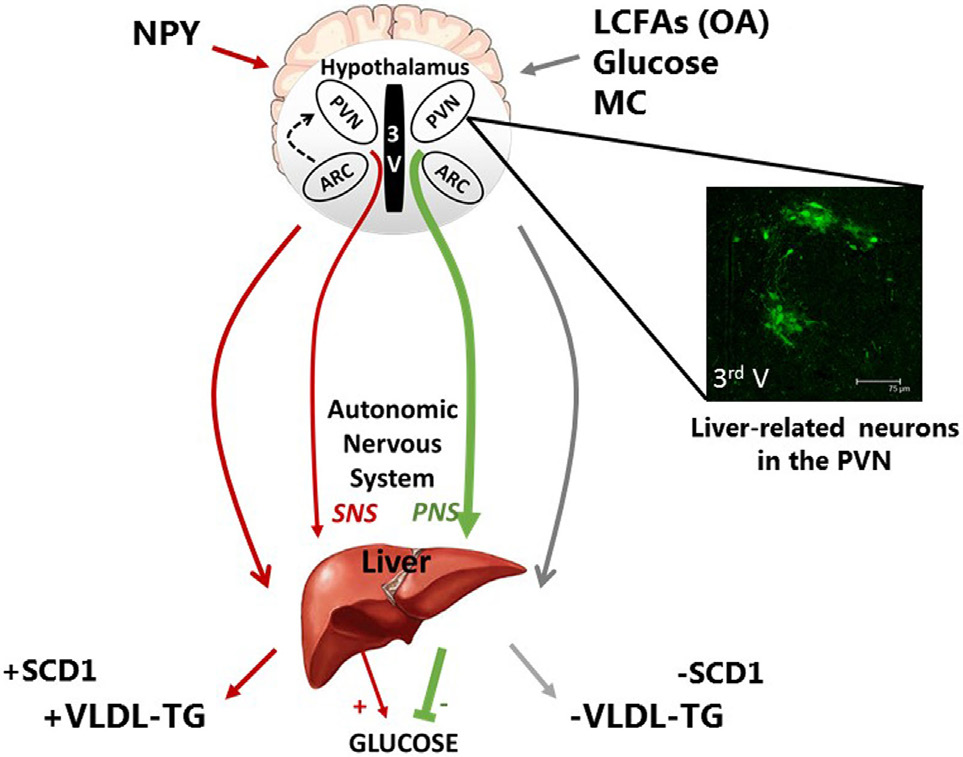
**Schematic representation of the neuronal regulation of hepatic carbohydrate and lipid metabolism**. Neurons of discrete hypothalamic nuclei including the paraventricular nucleus (PVN) and arcuate nucleus (ARC) within the mediobasal hypothalamus respond to neuronal inputs to regulate hepatic glucose and very low density lipoprotein (VLDL-TG) metabolism. The accumulation or application of long-chain fatty acids (LCFAs), such as oleic acid (OA), glucose, and melanocortin (MC) signaling act through the autonomic nervous system to signal nutritional plenty a reduced requirement for energy substrate mobilization, by inhibiting VLDL-TG secretion and hepatic glucose production (HGP). This is at least in part due to reduced stearol-CoA desatrate-1 (SCD1) expression. In contrast, hypothalamic NPY is indicative of hunger and nutritional deficiency and can increase energy substrate mobilization *via* an increase in VLDL-TG and SCD1 expression and HGP. The features of these fatty acid and nutritionally sensitive neurons are unclear; however, pseudorabies virus-152 labeling supports the notion that liver-related neurons in the PVN exist and may be involved in this autonomic regulation of hepatic metabolism.

This neurocircuitry is relevant to the pathogenesis of obesity, since numerous models of obesity and diabetes are characterized by elevated NPY. In a recent study, ICV administration of NPY or a selective NPY Y1 receptor agonist was shown to robustly elevate key genes involved in MUFA synthesis, such as stearol-CoA desatrate-1, and PL remodeling, such as ribosylation factor-1 (ARF-1) and lipin-1 (87). Importantly, these effects were attenuated following sympathetic denervation of the liver, supporting a model in which central NPY modifies hepatic PL and VLDL *via* the sympathetic signaling to the liver (87). Although these findings suggest that central lipid sensing may be implicated in the regulation of hepatic lipid homeostasis (see Figure 1), the direct mechanisms remain elusive. Nonetheless, a number of recent studies highlight the role of central FAs in hepatic lipid homeostasis. For example, central administration of PA resulted in impaired leptin signaling and pro-inflammatory response in the MBH and PVN (88). Furthermore, this was coupled with blunted leptin-induced changes in hepatic gluconeogenesis, glucose transportation, and lipogenesis (88). In a recent report, Yue and colleagues have shown that infusion of OA directly into the MBH activates a PKC-δ to K_ATP_ channel axis, which suppresses VLDL-TG secretion in rats (89). Moreover, this signaling requires DVC and hepatic innervation, highlighting a novel MBD-DVC neurocircuitry that mediates MBH FA sensing and hepatic lipid homeostasis (89). These findings are one step closer to the development of novel therapies that lower VLDL-TG secretion and restore lipid homeostasis in metabolic disorders; however, there is considerably more to learn regarding differential function of other hypothalamic neurons and their role in the autonomic regulation of hepatic metabolism.

## Liver-Related Neurons in the Hypothalamus

Recent findings support the existence of liver-related neurons in key hypothalamic nuclei; therefore, we can speculate that these liver-related neurons are the link between central lipid sensing and hepatic lipid metabolism. The control of hepatic functions by the ANS is well known. In general, beside the abovementioned examples, sympathetic stimulation of the liver enhances endogenous glucose production and glycogenolysis, while the parasympathetic nerves are responsible for inhibiting glucose production and promoting glucose storage (90–92). Preganglionic neurons are located in the spinal cord and brainstem, respectively, and transmit the information through the sympathetic and parasympathetic nerves. These autonomic motor neurons receive information from preautonomic neurons, which are located in higher brain areas and crucial for integration of brain signals (93, 94). Therefore, identifying the location of liver-related neurons and determining their cellular and molecular properties would be crucial to the understanding of brain–liver circuit.

Within the hypothalamus, the ARC, VMH, DMH, LH, and PVN are well recognized for their involvement in the regulation of a variety of metabolic functions of the body, and in particular the control of hepatic metabolism (95–101). Electrical and chemical stimulation have been feasible methods to establish direct connections between the hypothalamus and sympathetic neurons in the spinal cord or parasympathetic neurons in the brainstem and to demonstrate the importance of the autonomic control in hepatic functions (98, 102–105). On the other hand, establishing the location of premotor inputs to the sympathetic and parasympathetic motor neurons has been more challenging. Anterograde and retrograde tracers in combination with histochemical studies provided valuable information on the connections between the brain and liver (106), and the development of retrograde viral tracers opened new avenues to dissect the brain–liver pathway. Currently, transsynaptic neurotropic viruses are very valuable tools for identification of synaptic connections and neural networks. Among the neurotropic viruses, pseudorabies viruses (PRVs) are often used for circuit analysis and revealing organization of the nervous system (107–110). PRVs, such as PRV-152, an attenuated viral strain driving the expression of EGFP, are reliable and effective transsynaptic tracers, and numerous publications reported consistent organ-specific labeling of neurons (111–115). The spread of PRV-152 is strictly retrograde across synapses, and the virus is not capable of assembling in axons or glia; therefore, labeling of neurons not specific to the liver is unlikely, as has been shown (110, 116–118).

Polysynaptic neural connections between the brain and liver were identified in rodents using PRVs (118–120). PRV labeling was observed in the spinal cord of rats 3 days following inoculation, whereas at this time point no labeling was detected in parasympathetic nuclei (119). Labeled neurons in the dorsal motor nucleus of the vagus (DMV) were observed 4–5 days after inoculation of the liver. At this time point, liver-related neurons were also detected in the brainstem including the ventrolateral medulla, NTS, raphe pallidus, and few neurons were identified in the hypothalamic PVN and LH (119). Longer survival time provided labeling of liver-related neurons in nuclei connected to the PVN including the medial preoptic area, anterior hypothalamic area, and ARC. PRV-labeled cells were also present in the VMH, suprachiasmatic nuclei, central amygdala, and bed nucleus of stria terminalis (119). This study demonstrated that PRV provides reliable identification of polysynaptic sympathetic and parasympathetic pathways to the liver (113, 115, 118, 120).

Despite the identification of liver-related neurons in the CNS, labeling with PRV does not distinguish between sympathetic and parasympathetic liver-related neurons in higher brain areas. In order to identify sympathetic- or parasympathetic liver-related hypothalamic neurons in rats, PRV inoculation was combined with hepatic sympathectomy or parasympathectomy (121, 122). Following sympathetic denervation of the liver, parasympathetic liver-related neurons were identified in the brainstem DMV and nucleus ambiguus (122). Medium length survival time (~4 days) resulted in labeling of additional brainstem areas (e.g., NTS, area postrema) and hypothalamic nuclei including PVN, LH, DMH, ARC, and others (122). Retrograde labeling following parasympathectomy identified pre-sympathetic liver-related neurons in the PVN, medial preoptic area, anterior hypothalamic area, DMH, ARC, VMH, and SCN (121). These studies revealed that preautonomic PVN neurons project either to sympathetic or parasympathetic division, and the segregation of the neurons exists in other hypothalamic areas including LH and SCN suggesting functional specialization of preautonomic neurons controlling liver function. The segregation of the autonomic divisions was also supported by the observation that stimulation of PVN resulted in hyperglycemia largely due to sympathetic activation of the liver (121). On the other hand, we have to note, that viral injection into peripheral organs causes infection of nerve terminals innervating both vascular and non-vascular tissues (123). PRV injected into the liver is taken up by nerve endings near hepatocytes and sinusoidal cells, and we cannot distinguish between neurons innervating liver function or hepatic vasculature at the injection site. Therefore, it is likely that the PRV-labeled pre-sympathetic neurons contribute to both vasomotor and non-vasomotor sympathetic innervation of the liver.

Our current knowledge regarding the role and cellular properties of liver-related hypothalamic neurons is somewhat limited. Earlier studies suggested that the VMH is involved in the sympathetic control of the liver, whereas the LH plays role in the parasympathetic control of the liver (91, 124, 125). The PVN was also shown as an important, integrative center for the regulation of sympathetic and parasympathetic pathways to the liver (121, 126–128). Hypothalamic action of metabolic signals including insulin, leptin, and FAs has been shown to control glucose homeostasis (58, 59, 76, 129–133); however, the cellular properties, the phenotype, or the involved neural circuits underlying the central control of hepatic functions are less defined.

Hypothalamic nuclei are heterogeneous containing different types of neurons, which are able to control multiple organ systems; therefore, identification of pathway-related neurons is crucial. Our laboratory used the retrograde PRV tracing technique, discussed above, to identify liver-related neurons in the PVN and to reveal synaptic properties of liver-related neurons in control and hyperglycemic conditions (113). The studies determined that liver-related neurons receive transient receptor potential vanilloid type 1 (TRPV1) containing inputs. TRPV1 is a non-selective cation channel and has been linked to the development and progression of type 1 diabetes mellitus and T2D (134), and recent reviews have discussed its role in diabetes mellitus and obesity in further detail (135, 136). The TRPV1-dependent excitation of liver-related PVN neurons was diminished in a hyperglycemic, insulin-deficient mouse model. Both *in vivo* and *in vitro* insulin replacement restored the TRPV1-dependent excitatory neurotransmission *via* PI3-kinase, PKC, and/or TRPV1 trafficking (113). Similarly, TRPV1 was shown to play role in the regulation of excitatory neurotransmission to motor neurons in the DMV and stomach-related neurons in the PVN (137, 138). Furthermore, potential interaction between leptin signaling and TRPV1 has been proposed in the brainstem (114). In addition, there is limited information about the neurochemical phenotype of liver-related neurons. A study by Stanley and coworkers identified liver-related hypothalamic neurons using PRV and showed that a subpopulation of liver-related neurons co-localized with oxytocin and CRH in the mouse PVN (118). In the LH, a subset of MCH and orexin neurons was shown to be liver related. In the ARC, a subpopulation of POMC neurons but not NPY-expressing neurons were labeled with PRV, indicating that they are part of the brain–liver pathway (118).

Despite the scientific advances in our understanding of liver-related neurons, their precise role in the autonomic regulation of hepatic lipid metabolism remains to be determined. Although there is clear evidence that increased sympathetic outflow to the liver may increase VLDL-TG production, resulting in increased systemic FA availability, it remains to be determined whether the liver-related neurons of the hypothalamus are indeed FA sensitive, or indeed whether the FA-sensing neurons of the hypothalamus are liver related. It is plausible to suggest that liver-related neurons that direct hepatic triglyceride metabolism may express key lipid processing factors involved in lipid transport, e.g., CD36, FATP1 and/or TG metabolism, e.g., LPL. However, a more detailed understanding of these fundamental autonomic processes is needed in order to identify novel therapeutic targets that may halt the development of lipid-related disorders.

## Summary, Implications, and Interventions

The findings summarized in this review highlight the role of central lipid sensing in the regulation of systemic metabolism, including food intake, body weight, and hepatic glucose and lipid metabolism. Since increased HGP is a key factor in the development of glucose intolerance, understanding the neuronal mechanisms that drive the central regulation of HGP may be key to the development of novel therapeutic strategies that prevent hyperglycemia and the development of type 2 diabetes. In addition, our increased understanding of the hypothalamic control of TG metabolism highlights the potential utility of specifically modulating sympathetic nervous system activity toward the liver reduces VLDL-TG secretion and combat hypertriglyceridemia (139). The clinical relevance of this strategy is highlighted by a recent north European human cohort, in which elevated sympathetic nervous system activity was associated with features of the MetS, such as elevated circulated VLDL-TG (140). While current interventions for hypertriglyceridemia are aimed at reducing circulating FAs by increasing FA uptake from the plasma, an alternative approach to effectively lower TG could be though decreasing VLDL-TG production by the liver. This is the case with GLP-1 receptor agonists, which have been shown to decrease both hepatic lipogenesis (141) and VLDL-TG production (142). Based on the literature outlined in this review, interventions targeting specific liver-related or FA-sensitive neurons in of the hypothalamus could have a similar impact on systemic metabolism, and we recommend that the identification of the features of these neuronal populations should be the subject of intensive research focus.

## Author Contributions

KB, AZ, and RE wrote and edited the manuscript.

## Conflict of Interest Statement

The authors declare that the research was conducted in the absence of any commercial or financial relationships that could be construed as a potential conflict of interest.
